# Prevalence and molecular characterization of multi-resistant *Escherichia coli* isolates from clinical bovine mastitis in China

**DOI:** 10.1080/10495398.2024.2322541

**Published:** 2024-03-13

**Authors:** Hongxia Zhao, Hailan Ma, Chen Song, Shuting Fan, Hongliang Fan, Weiguang Zhou, Jinshan Cao

**Affiliations:** a Department of Pharmacology and Toxicology, College of Veterinary Medicine, Inner Mongolia Agricultural University, Huhhot, PR China; b Middle East College of Beijing International Studies University, Beijing, PR China; c Inner Mongolia Yili Industrial Group Co. Ltd., Huhhot, PR China

**Keywords:** *Escherichia coli*, integrons, ESBLs, fluoroquinolone, resistance-determining regions

## Abstract

Different antibiotics are used to treat mastitis in dairy cows that is caused by *Escherichia coli (E. coli).* Antimicrobial resistance in food-producing animals in China has been monitored since 2000. Surveillance data have shown that the prevalence of multiresistant *E. coli* in animals has increased significantly. This study aimed to investigate the occurrence and molecular characteristics of resistance determinants in *E. coli* strains (*n* = 105) obtained from lactating cows with clinical bovine mastitis (CBM) in China. A total of 220 cows with clinical mastitis, which has swollen mammary udder with reduced and red or gangrenous milk, were selected from 5000 cows. The results showed 94.3% of the isolates were recognized as multidrug resistant. The isolates (30.5%) were positive for the class I integrase gene along with seven gene cassettes that were accountable for resistance to trimethoprim resistance (*dfrA17, dfr2d* and *dfrA1*), aminoglycosides resistance (*aadA1* and *aadA5*) and chloramphenicol resistance (*catB3* and *catB2*), respectively. The *bla*_TEM_ gene was present in all the isolates, and these carried the *bla*_CTX_ gene. A double mutation in *gyrA* (i.e., Ser83Leu and Asp87Asn) was observed in all fluoroquinolone-resistant isolates. In total, nine fluoroquinolone-resistant *E. coli* isolates were identified with five different types of mutations in *parC*. In four (44.4%) isolates, Ser458Ala was present in parE, and in all nine (9/9) fluoroquinolone-resistant isolates, Pro385Ala was present in gyrB. Meanwhile, fluoroquinolone was observed as highly resistant, especially in isolates with *gyrA* and *parC* mutations. In summary, the findings of this research recognize the fluoroquinolone resistance mechanism and disclose integron prevalence and ESBLs in *E. coli* isolates from lactating cattle with CBM.

## Introduction

In the twenty-first century, antibiotic resistance is a serious health issue and causing a huge problem for humans, animals and the environment and threatening life on the globe. The phenomenon occurs when pathogenic microbes are nonresponsive to the killing or inhibitory property of standard doses of antimicrobials. The cow and buffalo take a significant share of the antibiotic resistance for being reservoirs of the resistant strains. Different bacteria cause various bacterial diseases in animals such as mastitis. In China, dairy cows, buffaloes, goats and sheep play a crucial role in the economy of the country and also fulfil the requirements of the local herdsman. These animals are reared for the production of milk, meat, wool and hair. In China, many dairy cows and goat farms are available and produce millions of tons of milk annually. Due to the high production of milk, these animals are easily susceptible to viral, bacterial and parasitic infections which alter the health of the animals as well as reduce the milk production and alternatively affect the economy of the country. Dairy cattle are more susceptible to mastitis than other species such as buffalo, goat and sheep. Bovine mastitis (BM) is the main health problem in high-producing cows in dairy farms. The common use of antimicrobial agents in disease treatment can potentially lead to the development of antimicrobial-resistant bacteria.^1^

BM is a prevalent disease with an occurrence rate between 23% and 40% in dairy cow farms.^2^ BM causes many economic losses in most dairy farms, including treatment, discarded milk, labour, fatality costs, repeated cases of mastitis, decreased milk yield and milk quality changes.^3^^,^^4^ Numerous microorganisms associated with cases of BM have been isolated. The most frequently isolated pathogens that resulted in clinical BM (CBM) in China are *Escherichia coli, Klebsiella spp., coagulase-negative staphylococci*, *Streptococcus dysgalactiae* and *Staphylococcus aureus.*^5^^,^^6^ CBM caused by *E. coli* ranges from a moderate disease with a short duration to serious, per acute and life-threatening diseases. Appropriate antimicrobial therapy can effectively alleviate the symptoms and reduce the risk of complications of CBM. To date, some antimicrobial agents, i.e., beta-lactams, tetracyclines, macrolides, aminoglycosides and fluoroquinolones, have been commonly used to treat CBM in China.^7^ Among these drugs, the most widely used antibiotics with convincing evidence of their effectiveness in treating *E. coli* mastitis are fluoroquinolones and cephalosporins, particularly 3rd and 4th generation drugs.^8^ With the widespread use of antimicrobials, multidrug resistant *E. coli* isolates have emerged in many dairy farms.^1^^,^^9^

Integrons are found in the isolates of *E. coli* that are multidrug resistant from lactating cattle. As natural genetic elements, Integrons can capture, integrate and mobilize antibiotic-resistant gene cassettes. The majority of antibiotic-resistant genes in clinical isolates of Gram-positive bacteria are Class 1 integrons.^10^^,^^11^ There are already more than 130 gene cassettes known, and the encoded products would make bacteria resistant to practically all antibiotics.^12^ According to previous research, class I integrons were prevalent in clinical *E. coli* isolates, as well as contributing to the development of antimicrobial resistance in BM isolates.^13^ A latent pool of antimicrobial resistance genes exists in *E. coli* isolates from animals carrying class I integrons, while integrons can be found on plasmids or in transposons, facilitating the transfer of antibiotic resistance genes among bacteria.^14^ In recent years, extended-spectrum ß-lactamases (ESBLs) have been increasingly detected among Gram-negative bacteria isolated from animals. They are mediated by the *bla_SHV_*, *bla_TEM_* and *bla_CTX-M_* genes in Gram-negative bacteria.^15^ Based on studies from different hospitals, the CHINET national bacterial surveillance project found a 56.2% detection rate of ESBL-producing *E. coli* in 2009.^16^ ESBLs and class 1 and class 2 integrons are regularly recovered from domestic livestock, demonstrating that animals can serve as reservoirs for resistance genes.^1^

The antibiotic class known as fluoroquinolones is extremely effective and has several benefits, such as high oral absorption, wide volume of distribution and broad-spectrum antibacterial action,^17^ and is frequently used for dealing with both Gram-negative and positive bacterial infections in clinics. Fluoroquinolones are considered a critically important antibiotic by the World Health Organization (WHO) due to their clinical importance in the field of human and animal medicine.^18^ A significant public health risk arises from the consistent use of fluoroquinolones to treat livestock.^19^ Fluoroquinolone resistance can be caused by various phenomena, of which numerous point mutations in the quinolone resistance-determining region (QRDR) of the DNA gyrase (gyrA and gyrB) and topoisomerase IV enzymes (parC and parE) are more important.^20^ Chromosomal mutations in QRDRs resulted in amino acid replacement, structurally altering the target protein and later altering the binding affinity of the drug.^21^ In addition, the mechanism of fluoroquinolone resistance also included plasmid-mediated quinolone resistance (PMQR). A major facilitator superfamily-type quinolone efflux pump qepA gene is an encoded gene, an aminoglycoside acetyltransferase, aac(6′)-Ib-cr gene, which confers reduced susceptibility to ciprofloxacin, and a QNR gene, which protects DNA gyrase and topoisomerase IV from quinolone inhabitation.^22^ Although PMQR genes only provide moderate fluoroquinolone resistance, they can help select mutation in topoisomerase and gyrase genes, leading to greater levels of fluoroquinolone resistance.^23^

Our study elucidate the molecular characteristics and prevalence of antibiotic resistance in *E. coli* strains from CBM in order to produce baseline data that will be used to assess future antimicrobial resistance risk. In addition, our study investigates why fluoroquinolone-resistant *E. coli* are found in CBM cases following over 20 years of fluoroquinolone use in Chinese veterinary medicine.

## Materials and methods

### Selection of clinical mastitis cases in dairy cows and sample collection

The methods and all the protocols used in this research were permitted by the related rules and regulations of the scientific research academic ethics committee, Inner Mongolia Agricultural University ([2020]086) that approved the study. All authors complied with the above guidelines and regulations. The clinical mastitis cases were selected in five dairy farming bases around Hohhot, China’s ‘milk capital’, of China. A total of 220 cows with clinical mastitis and swollen mammary glands with reduced and red or gangrenous milk were selected from 5000 cows. The owners of the five dairy farms agreed to carry out the study, and the milk of cows suffering from clinical mastitis was collected after obtaining the owner’s permission. Some milk samples were collected from cows with clinical mastitis at postpartum. The other samples were collected during the whole lactation. From the samples, there were 143 mild mastitis cases, 55 moderate mastitis cases and 22 severe mastitis cases. Mild mastitis cases (Grade 1): The breast is not abnormal on palpation, and the milk has visible changes, thinning of milk, somatic cell count of more than 500,000/mL and strong positive CMT test. Moderate mastitis cases (Grade 2): there are more serious pathological changes in the breast tissue, and the affected udders quarters are red, swollen, hot and painful. The milk has visible changes, turning grey or watery, with clots. However, the dairy cows with moderate mastitis have no systemic symptoms. Severe mastitis (Grade 3): there are more serious pathological changes in the breast tissue, and the affected udder quarters are red, swollen, hot and painful. The milk has visible changes, turning grey or watery. There are clots in the milk, and dairy cows with severe mastitis have obviously systemic symptoms. The cows’ udders and nipples were washed with warm water, and the nipples were sterilized with iodophor. An aliquot of 10 mL of milk was collected from each udder quarter of the dairy cows with clinical mastitis and stored in sterilized test tubes. Samples of milk were brought to the laboratory in ice and analysed within 12 h of collection. About 252 milk samples were collected between 2015 and 2020.

### Bacterial isolation and confirmation

Eosin methylene blue agar was used and 10 µL of milk sample was streaked and incubated for 18–24 h at 37 °C. Presumptive *E. coli* colonies with the purple-dark green metallic luster were further confirmed with gram staining as per instruction of the manufacturer. Vitek system (bioMerieux) was used. Then*, uida* gene was identified by polymerase chain reaction (PCR) to confirm *E. coli* and *uida* gene is a specific gene of *E. coli* to confirm the Identification. PCR was used to further biochemically verify the *E. coli* isolate according to the protocol mentioned earlier.^1^

### Antibacterial susceptibility determination

A total of 22 antibiotic agents were bought from the CIVDC China and were used in current research. The categories of antimicrobials are as follows: aminoglycosides (kanamycin, amikacin, streptomycin and gentamycin), penicillins (ampicillin and amoxicillin), cephalosporins (ceftiofur, cefalotin and cefazolin), tetracyclines (oxytetracycline, doxycycline and oxytetrcycline), colistin, phenylpropanol (chloramphenicol and florfenicol), folic acid antagonists (sulphadiazine, sulphamethoxydiazine and trimethoprim), fluoroquinolones (norfloxacin, ciprofloxacin, lomefloxacin and enrofloxacin). Penicillins and cephalosporins both belong to β- Lactamides. Multidrug resistance (MDR) was defined as acquired nonsusceptibility to at least one agent in three or more antimicrobial categories.^24^ According to the rules and regulations of the Clinical and Laboratory Standard Institutes 2015 performance Standards for susceptibility testing, the antibiotic drugs were dissolved and diluted.^25^ Every day, new dilutions of all substances were made.

On a 96-well polystyrene plate with a ‘U’ type bottom, Minimal inhibitory concentrations (MICs) for *E. coli* isolates were calculated by the microdilution protocol 2015 clinical and laboratory standards were used to interpret the findings.^25^ The quality control strain for bacterial isolation and drug susceptibility testing was standard strain ATCC25922.

### Molecular epidemiological analysis of *E. coli* ESBLs genes and class I, II and III integrons

The ESBLs genes were detected by multiplex PCR. First, the DNA templates including genomic and plasmid DNA from *E. coli* isolates were prepared using Bacteric DNA Kit (Promega, Madison, WI) and MiniBest Plasmid Purification Kit. The major members of the β-lactamase gene family (i.e., the *bla*_TEM_, *bla*_SHV_ and *bla*_CTX_ genes) were amplified by multiplex PCR. PCR products were analyzed by electrophoresis on 1% agarose gels stained with ethidium bromide.^26^

PCR was used to detect the integrons in all of the isolates. The PCR settings employed to find the gene cassettes and class I integrons have previously been disclosed. The PCR amplification consisted of initial denaturation at 95 °C for 5 min followed by 35 cycles of denaturation at 94 °C for 30 s, class II integron annealing at 52 °C for 30 s, class III integron annealing at 50 °C for 30 s and extension at 72 °C for 40 s, followed by a final extension at 72 °C for 5 min.^12^ Through electrophoresis in 1.0% agarose gels, the amplification products were separated, and they were then visible in ultraviolet light. Primer sets covering integron-variable areas were used to create the amplifications, which were then gel-purified and cloned into the pGEM-T Easy vector (Promega) using conventional techniques. All reactions were repeated three times. To determine whether different isolates carried identical integrons, the amplicons of similar sizes were compared by restriction fragment length polymorphism (RFLP) analysis using the enzyme EcoRI and standard methods,^1^ If the amplicons from two strains yielded the same RFLP pattern, the integrons were considered identical. If the PCR product contained a different RFLP pattern, the new product was also sequenced.

### QRDR gene mutation analysis

The QRDRs of gyrA, parC, gyrB and parE genes were amplified from nine strains of fluoroquinolone-resistant *E. coli* by PCR. Using a Wizard Genomic DNA Purification Kit from Promega, genomic DNA was extracted from quinolone-resistant *E. coli* isolates. Employing previously defined PCR primers and amplification conditions, the QRDRs of the genes gyrA, parC, gyrB and parE were amplified. The PCR protocol was done as mentioned in our earlier study Zhao et al. (2014)^1^ and primer sequences and PCR product sizes of genes for antimicrobial resistance are mentioned in Table 1. The amplification results have been isolated by electrophoresis on 1.0% agarose gel and seen in ultraviolet light.

**Table 1.** Primers sequences and PCR product sizes of genes for antimicrobial resistance.

Primers	Primer sequence (5′–3′)	Size of amplification (bp) Ref.
Intl1	F: CCTCCCGCACGATGATC	280 [12]
	R: TCCACGCATCGTAAGGC	
Intl2	F: CGTGCTGGAGGGAAAGAC	207 [12]
	R: CATGACGGTAAGGGTGGG	
Intl3	F: AGTGGGTGGCGAATGAGTG	600 [12]
	R: TGTTCTTGTATCGGCAGGTG	
5′-CS	GGCATCCAAGCAGCAAG	Uncertain [1]
3′-CS	AAGCAGACTTGACCTGA	
*bl*a_SHV_	F: TCTCCCTGTTAGCCACCCTG	593 [1]
	R: CCACTGCAGCAGCTGCCGTT	
*bla*_TEM_	F: GTATCCGCTCATGAGACAATA	717 [1]
	R: AGAAGTGGTCCTGCAACTTT	
*bla*_CTX_	F: ACAGCGATAACGTGGCGATG	443 [1]
	R: TCGCCCAATGCTTTACCCAG	
*gyrA*	F: GAGGGATAGCGGTTAGATGAGC R: CCGTTCACCAGCAGGTTAGG	525 [1]
*parC*	F: AATGAGCGATAGGCAGAGCG R: TTGGCAGACGGGCAGGTAG	487 [1]
*gyrB*	F: GCCTTTCTTCACTTTGTACAGCG R: GTGACGGCGGTACTCACCTG	794[1]
*parE*	F: CTGACCGAAAGCTACGTCAACC	892 [1]
	R: CGTTCGGCTTGCCTTTCTTG	
*qnrA*	F: CAACTTGAGTGGCCAATGCC	211 [27]
	R: GACTCCTTCGAGGTTGACCC	
*qnrS*	F: ACGACATTCGTCAACTGCAA	417[27]
	R: TAAATTGGCACCCTGTAGGC	
*qnrB*	F: GGMATHGAAATTCGCCACTG	264 [27]
	R: TTTGCYGYYCGCCAGTCGAA	
*qnrC*	F: GGGTTGTACATTTATTGAATC	447 [27]
	R: TCCACTTTACGAGGTTCT	
*qnrD*	F: CGSGATCAATTTACGGGGAATA	582 [27]
	R: AACAAGCTGAAGCGCCTG	
*qepA*	F: GCAGGTCCAGCAGCGGGTAG	199 [28]
	R: CTTCCTGCCCGAGTATCGTG	
*aac(6′)-Ib-cr*	F: TTGCGATGCTCTATGAGTGGCTA	482 [28]
	R: CTCGAATGCCTGGCGTGTTT	

### PMQR genes detection by PCR

The availability of the PMQR genes *qnrA, qnrS, qnrB, qnrC, qnrD, qepA* and *aac(6′)-Ib-cr* was identified according to the previously mentioned.^27^

### Sequencing of the DNA and data analysis

Nucleotide sequences were sequenced with ABI PRISM Big Dye Primer Cycle Sequencing Ready Reaction Kit and ABI 377 DNA auto-sequencing machine by using T7 and SP6 sequence primers (Perkin-Elmer Company, Waltham, MA). For analysis of the data, the software DNASTAR (DNASTAR, Inc., St. Madison, WI) and the programme NCBI-BLAST (www. ncbi.nlm.nih.gov) were used. The nucleotide sequence data generated in this study have been submitted to GenBank under accession numbers X06373, AE000447, M58408, M58409, EF488368, EF488369 and EF488370.

## Results

### Antimicrobial susceptibility

In Table 2, the results of the antimicrobial susceptibility of 105 *E. coli* isolates with CBM to 22 antimicrobial agents are presented. As shown in Table 2, greater resistant incidence rates were shown for sulphamethoxydiazine (100%), sulphadiazine (99.0%), tetracycline (76.2%), oxytetracycline (71.4%), ampicillin (69.5%), cefazolin (62.9%) and amoxicillin (60.0%). However, most of the isolates were susceptible to fland amoxicilli. The isolates’ resistance rates of the different antibiotics such as ciprofloxacin, lemofloxicin, norfloxacin and norfloxacin were 28.6%, 27.6%, 27.6% and 27.6%, respectively. The high susceptibility rate of *E. coli* strains to ceftiofur was 89.5%, followed by colistin (74.3), amikacin (69.5), kanamycin (65.7%), streptomycin (63.8%), gentamicin (63.8%), chloramphenicol (55.2%) and florfenicol (54.3%).

**Table 2.** *In vitro* susceptibilities of 105 *E. coli* strains isolated from CBM to 22 antimicrobial agents.

Antibacterial agents	No. (%) of resistant strains^a^		
	R	I	S
Streptomycin	38 (36.2)	—	67(63.8)
Kanamycin	34 (32.4)	2 (1.9)	69 (65.7)
Gentamicin	31 (29.5)	6 (5.7)	67 (63.8)
Amikacin	26 (24.8)	6 (5.7)	73 (69.5)
Amoxicillin	63 (60.0)	6 (5.7)	36 (34.3)
Ampicillin	73 (69.5)	3 (2.9)	29 (27.6)
Cefazolin	66 (62.9)	10 (9.5)	29 (27.6)
Ceftiofur	10 (9.5)	1 (1)	94 (89.5)
Cefalotin	34 (32.4)	30(28.6)	41 (39.0)
Tetracycline	80 (76.2)	7(6.7)	18 (17.1)
Doxycycline	50 (47.6)	13 (12.4)	42 (40.0)
Oxytetracycline	75 (71.4)	0 (0)	30 (28.6)
Colistin	27 (25.7)	—	78 (74.3)
Chloramphenicol	43 (41.0)	4 (3.8)	58 (55.2)
Florfenicol	48 (45.7)	—	57 (54.3)
Enrofloxacin	29 (27.6)	3 (2.9)	73 (69.5)
Norfloxacin	29 (27.6)	7 (6.7)	69 (65.7)
Lomefloxacin	29 (27.6)	11 (10.5)	65 (61.9)
Ciprofloxacin	30 (28.6)	3 (2.8)	72 (68.6)
Sulphadiazine	104 (99.0)	—	1 (1.0)
Sulphamethoxydiazine	105 (100)	0 (0)	0 (0)
Trimethoprim	105 (100)	0 (0)	0 (0)

^a^R: resistant; I: intermediate; S: susceptible

**Figure 1.**
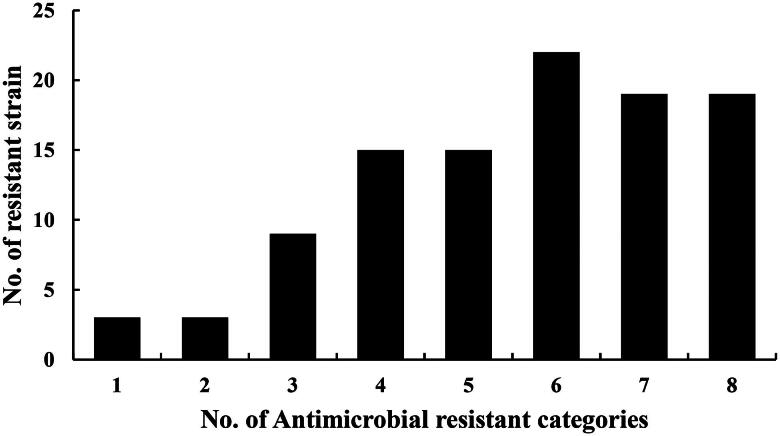
Multidrug resistant levels of *E. coli* isolates from CBM.

**Figure 2.**
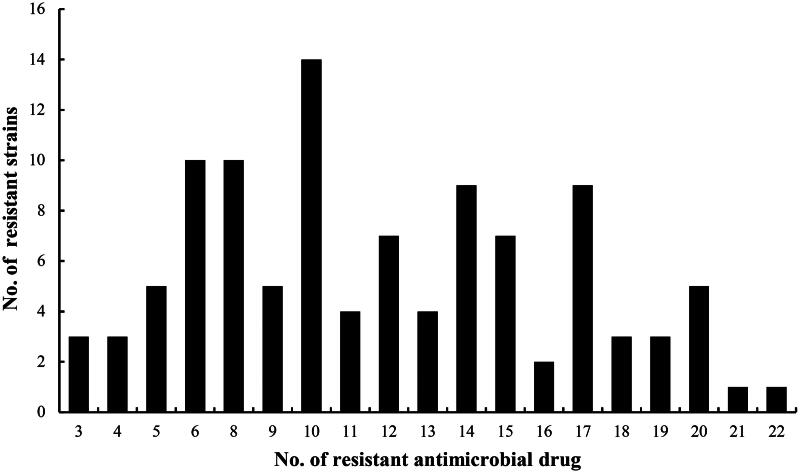
Antimicrobial resistant profile of *E. coli* isolates from CBM.

As shown in Figure 1, the results showed 94.3% (99/105) of the isolates were recognized as multidrug-resistant and only 6 isolates were resistant to one or two antimicrobial categories. The resistant phenotypes of the different multidrug resistant isolates were different. But most of them were resistant to trimethoprim, sulphamethoxydiazine, sulphadiazine, tetracycline, oxytetracycline, ampicillin, amoxicillin and cefazolin. A total of six strains of *E. coli* were found to have MDR three or more antimicrobial categories when 105 isolates were tested against 22 antimicrobial drugs. Seventy-five isolates were resistant to more than five antibiotic categories of drugs mentioned earlier, accounting for 71.4%. From Figure 2, there were 69 isolates resistant to more than 10 antibiotic drugs used in the experiments, and account for 65.7%. The results of susceptibility of antimicrobial displayed that those 15 isolates were simultaneously resistant to ciprofloxacin, enrofloxacin, norfloxacin and lomefloxacin, confirmed as fluoroquinolone-resistant *E. coli* isolates.

### Class 1 integrons and ESBLs genes characterization

A total of 30.5% (32/105) of the isolates were noticed for the existence of *intI1* (class I integrase gene), which was the indicator for class I integrons. None of the isolates harboured class II integrons and class III integrons. Thus, the integron-borne gene cassettes were cloned and sequenced. As shown in Table 3, the integrons contained one to three gene cassettes and the combinations of these gene cassettes.

**Table 3.** Antibiotic resistance phenotypes and resistance-determining regions in *E. coli* isolates from dairy cattle with CBM.

Strain number	Resistance phenotypes^a^	Gene cassettes of class 1 Integron^b^		β-Lactamase gene^c^
10	STR, AMC, TET, DOX, OXY, AMP, SMD, ENR, NOR, LOM, CIP, CET, SAD, TMP	Integrase1	*dfr2d-catB3-aadA1*	*bla*_TEM_
22	STR, CEZ, CHL, AMC, AMP, FOR, TET, DOX, OXY, SAD, SMD, TMP	Integrase1	*dfrA1-aadA1*	*bla*_TEM_
23	STR, KAN, GEN, AMC, AMP, CEZ, CET, TET, DOX, OXY, CST, CHL, ENR, NOR, LOM, CIP, SAD, SMD, TMP	Integrase1	*dfrA1-catB2-aadA1*	*bla*_TEM,,_ *bla*_CTX_
25	STR, KAN, AMK, CEZ, CST, CHL, AMP, TET, OXY, CET, SAD, SMD, TMP, AMC	Integrase1	*aadA1*	*bla*_TEM_
26	CST, CHL, AMP, TET, OXY, AMC, FOR, SAD, SMD, TMP	Integrase1	*aadA1, dfrA1-aadA1, dfrA17-aadA5*	*bla*_TEM_
27	STR, KAN, CEZ, CST, FOR, AMC, AMP, TET, DOX, OXY, SAD, SMD, TMP, ENR, NOR, LOM, CIP	Integrase1	*dfrA17-aadA5*	*bla*_TEM_
28	KAN, GEN, AMK, AMC, AMP, CEZ, TET, DOX, OXY, CST, CHL, ENR, NOR, LOM, CIP, SAD, SMD, TMP	Integrase1	*aadA1, dfr2d-catB3-aadA1, dfrA17-aadA5*	*bla*_TEM_
30	STR, CHL, DOX, OXY, FOR, SAD, SMD, TMP	Integrase1	*aadA1*	*bla_T_*_EM_
34	CEZ, CST, CET, FOR, CHL, TET, OXY, SAD, SMD, TMP	Integrase1	*dfrA17-aadA5*	*bla*_TEM_
35	CHL, FOR, CET, AMP, TET, DOX, OXY, SAD, SMD, TMP	Integrase1	*aadA1*	*bla*_TEM_
41	STR, KAN, GEN, AMK, AMC, AMP, CEZ, CET, TET, DOX, OXY, CST, CHL, FOR, ENR, NOR, LOM, CIP, SAD, SMD, TMP	Integrase1	*aadA1, dfr2d-catB3-aadA1*	*bla*_TEM,_ *bla*_CTX_
44	STR, KAN, GEN, AMK, AMC, AMP, CEZ, CET, TET, DOX, OXY, SAD, SMD, TMP	Integrase1	*dfrA17-aadA5*	*bla*_TEM_
45	STR, AMC, AMP, TET, DOX, OXY, SAD, SMD, TMP	Integrase1	*dfrA17-aadA5*	*bla*_TEM_
50	STR, KAN, GEN, AMK, AMC, AMP, CEZ, CET, TET, DOX, OXY, CST, CHL, FOR, SAD, SMD, TMP	Integrase1	*dfr2d-catB3-aadA1, dfrA17-aadA5*	*bla*_TEM,_ *bla*_CTX_
54	STR, KAN, GEN, AMC, AMP, CEZ, CEF, CET, TET, DOX, OXY, CST CHL, FOR, NOR, LOM, SAD, SMD, TMP	Integrase1	*aadA1*	*bla*_TEM,_ *bla*_CTX_
55	STR, AMC, AMP, TET, DOX, OXY, SAD, SMD, TMP	Integrase1	*aadA1, dfrA17-aadA5*	*bla*_TEM_
56	STR, KAN, GEN, AMK, CEZ, CET, AMP, TET, DOX, CST, OXY, SAD, SMD, TMP	Integrase1	*aadA1*	*bla*_TEM_
59	STR, AMK, CEZ, CST, CHL AMP, TET, OXY, SAD, SMD, TMP	Integrase1	*dfrA1- catB2-aadA1*	*bla*_TEM_
67	AMC, CHL, TET, OXY, AMP, CET, ENR, NOR, LOM, CIP, SAD, SMD, TMP	Integrase1	*aadA1, dfrA17-aadA5*	*bla*_TEM_
73	STR, KAN, GEN, CEZ, CET, AMC, AMP, TET, DOX, OXY, CHL, FOR, SAD, SMD, TMP	Integrase1	*dfrA1-aadA1*	*bla*_TEM_
81	STR, KAN, GEN, AMC, AMP, TET, OXY, CST, CHL, CIP, SAD, SMD, TMP	Integrase1	*aadA1,* *dfrA1- catB2-aadA1*	*bla*_TEM,_ *bla*_CTX_
82	STR, AMK, CEZ, CST, CHL, SAD, SMD, TMP	Integrase1	*dfrA1- catB2-aadA1*	*bla*_TEM_
84	STR, KAN, AMK	Integrase1	*aadA1*	*bla*_TEM_
85	STR, KAN, AMP, CEZ, OXY, CST, CHL, ENR, NOR, LOM, CIP, SAD, SMD, TMP	Integrase1	*dfr2d-catB3-aadA1*	*bla*_TEM_
86	CEZ, OXY, FOR, SAD, SMD, TMP	Integrase1	*dfrA17-aadA5*	*bla*_TEM_
87	CEZ, TET, AMP, OXY, ENR, SAD, SMD, TMP	Integrase1	*aadA1, dfrA1-aadA1, dfrA17-aadA5*	*bla*_TEM_
89	CEZ, TET, SAD, SMD, TMP	Integrase1	*aadA1*	*bla*_TEM_
93	CEZ, CEF, CST, TET, OXY, CHL, FOR, SAD, SMD, TMP	Integrase1	*dfrA17-aadA5*	*bla*_TEM_
94	STR, KAN, GEN, AMC, AMP, CEF, TET, DOX, OXY, FOR, NOR, SAD, SMD, TMP, NOR	Integrase1	*dfrA1-aadA1*	*bla*_TEM_
100	CEZ, CST, CHL, SAD, SMD, TMP	Integrase1	*dfr2d-catB3-aadA1*	*bla*_TEM_
103	KAN, CEZ, TET, DOX, OXY, CST, FOR, SAD, SMD, TMP	Integrase1	*dfrA17-aadA5*	*bla*_TEM_
104	STR, AMC, AMP, CEZ, TET, DOX, OXY, CHL, FOR, SAD, SMD, TMP	Integrase1	*dfrA17-aadA5, dfr2d-catB3-aadA1*	*bla*_TEM_

^a^STR: streptomycin; KAN: kanamycin; GEN: gentamicin; AMK: Amikacin; AMC: Amoxicillin; AMP: ampicillin; CEZ: cefazolin; CEF: ceftiofur; CET: cefalotin; DOX: doxycycline; TET: tetracycline; OXY: oxytetracycline; CST: colistin; SAD: sulphadiazine; SMD: sulphamethoxydiazine; TMP: Trimethoprim; CHL: chloramphenicol; FOR: florfenicol; ENR: enrofloxacin; NOR: norfloxacin; LOM: lomefloxacin; CIP: ciprofloxacin

^b^*IntegraseI*: *integrase gene*; *aadA1*: aminoglycoside-3′-adenylytransferase gene; *dfrA17-aadA5*: dihydrofolate reductase gene and aminoglycoside-3′-adenylytransferase gene; *dfrA1-aadA1*: dihydrofolate reductase gene and aminoglycoside-3′-adenylytransferase gene; *dfrA1-catB2-aadA1*: dihydrofolate reductase gene and chloramphenicol acetyltransferase gene and aminoglycoside-3′-adenylytransferase gene

^c^*bla_TEM_*: TEM-type ESBL gene; *bla_CTX_*: CTX-type ESBL gene

Five distinct kinds of gene cassette arrays were characterized. These were *aadA1, dfr2d-catB3-aadA1, dfrA1-catB2-aadA1, dfrA1-aadA1* and *dfrA17-aadA5,* respectively. Of them, *dfrA17–aadA5* (43.8%) was found to be the most prevalent gene cassettes among these isolates, followed by *aadA1* (34.4%), *dfr2d-catB3-aadA1* (21.9%), *dfrA1-aadA1*(15.6%), *dfrA1-catB2-aadA1*(12.5%). *E. coli* isolates (105) from CBM were nominated to determine ß-lactamase genes by PCR protocol. All of *E. coli* isolates were found to carry the *bla*_TEM_ gene (100%), but not the *bla*_SHV_ gene. There were five isolates carrying the *bla*_CTX_ gene. The isolates that carried the *bla_TEM_* and *bla_CTX_* genes were resilient to penicillin, aminoglycosides, sulphonamides, trimethoprim, tetracyclines, chloramphenicol and fluoroquinolones.

### Mutations in QRDR

The genes such as *par*C, *par*E, *gyr*A and *gyr*B, mutation were distinguished by sequencing in 15 fluoroquinolone-resistant *E. coli* isolates. As shown in Table 4, Ser83Leu, Asp87Asn, Ser83Leu and Asp87Asn substitutions were observed in GyrA. In *par*C, five dissimilar swaps were identified in all fluoroquinolone-resistant isolates. Among them, the most common point substitution in the strains was Ser58Ile (80%), followed by Arg138Val (33.3%), Asn141Lys (26.7%), Arg138Asp (13.3%) and Pro140Thr (13.3%). Six isolates were found to have one substitution in ParE (i.e., Ser458Ala), and all of the fluoroquinolone-resistant isolates had the substitution Pro385→Ala in GyrB. Then, the relationship between gene mutations and drug-resistance phenotypes in fluoroquinolone-resistant strains was analyzed. A certain correlation was revealed between the number of mutations mostly identified in the QRDRs region of the *gyrA, parC* and *parE* genes and the rising MICs of fluoroquinolones (Table 4). Four isolates with a ciprofloxacin MIC of 4–8 µg/mL had no one or one point mutation in the QRDRs region of the *gyrA,* and *parC* genes, such as the isolates #80, #82, #90 and #105. Two isolates (#1 and #40) had a ciprofloxacin MIC of 16 µg/mL, harbouring one- or two-point mutations in these genes. Five isolates (#27, #62, #69, #73 and #86) with a ciprofloxacin MIC of approximately 32 µg/mL had three and five mutations in the *gyrA*, *parC* and *parE* genes. All isolates with high-level ciprofloxacin resistance (≥64 µg/mL) carried five or six mutations in these genes. Therefore, there was a correlation between the number of mutations in QRDR and increased fluoroquinolone MICs. Fluoroquinolone resistance at a high degree was observed, especially in isolates with both *gyrA* and *parC* mutations. However, the *gyrB* gene’s QRDR mutation was present in all of the fluoroquinolone-resistant isolates.

**Table 4.** Fluoroquinolone susceptibility and substitutions in GyrA, GyrB, ParC and ParE QRDRs in *E. coli* isolates from dairy cattle with CBM.

Isolate	ENR^a^	NOR^b^	CIP^c^	LOM^d^	GyrA^e^	ParC^f^	GyrB^g^	ParE^h^	Gene cassettes of class 1 Integron^i^		**β-Lactamase gene** ^j^
1	8	16	16	8	S83L	S58I	P385A				
9	64	32	64	32	S83L, D87N	S58I, R138V,	P385A	S458A			
14	64	32	64	32	S83L, D87N	S58I, R138D,	P385A				
27	64	32	32	16	D87N	S58I, P140T,	P385A	S458A	Integrase1	*dfrA17-aadA5*	*bla*_TEM_
40	16	32	16	16		S58I,	P385A				
61	16	32	64	16	S83L, D87N	S58I, N141K	P385A	S458A			
62	16	16	32	16		R138V, N141K	P385A	S458A			
69	8	32	32	16	S83L, D87N	S58I	P385A				
73	16	64	32	16	S83L, D87N	S58I, R138D	P385A	S458A	Integrase1	*dfrA1-aadA1*	*bla*_TEM_
80	8	16	4	16		R138V	P385A				
86	16	64	32	16	S83L, D87N	S58I, R138V	P385A		Integrase1	*dfrA17-aadA5*	*bla*_TEM_
96	16	32	8	16		S58I	P385A				
98	16	32	8	16		R138V, N141K	P385A	S458A	Integrase1	*dfr2d-catB3-aadA1*	*bla*_TEM_
100	32	8	64	16	S83L, D87N	S58I, P140T, N141K	P385A				
105	8	16	4	16		S58I					

^a^ENR: enrofloxacin minimal inhibition concentration ≥8 resistance

^b^NOR: norfloxacin minimal inhibition concentration ≥16 resistance

^c^CIP: ciprofloxacin minimal inhibition concentration ≥4 resistance

^d^LOM: lomefloxacin minimal inhibition concentration ≥8 resistance

^e^Number of amino acid substitution sites: S83I: substitution of serine to leucine at amino acid 83; D87N: aspartic acid to asparagine

^f^Number of amino acid substitution sites: S58I: serine to isoleucine; R138V: arginine to valine; R138D: arginine to aspartic acid; P140T: proline to threonine; N141K: aspartic acid to lysine

^g^Number of amino acid substitution sites: P385A: proline to alanine

^h^Number of amino acid substitution sites: S458A: serine to alanine

^i^*IntegraseI*, *integrase gene*; *aadA1*: aminoglycoside-3′-adenylytransferase gene; *dfrA17-aadA5*, dihydrofolate reductase gene and aminoglycoside-3′-adenylytransferase gene; *dfrA1-aadA1*: dihydrofolate reductase gene and aminoglycoside-3′-adenylytransferase gene; *dfrA1-catB2-aadA1*: dihydrofolate reductase gene and chloramphenicol acetyltransferase gene and aminoglycoside-3′-adenylytransferase gene

^j^*bla_TEM_*: TEM-type ESBL gene; *bla_CTX_*: CTX-type ESBL gene

### Plasmid-mediated quinolone resistance

The 15 fluoroquinolone-resistant isolates from CBM were determined for the availability of PMQR mechanisms. No PMQR genes were found in these fluoroquinolone-resistant *E. coli* isolates from CBM.

## Discussion

BM is a crucial problem for dairy farmers which affects the production performance of the dairy cows and also affects the health and economy.^29^ The cornerstone of this disease’s treatment is antibiotic therapy, but its therapeutic efficacy is constrained by the rising prevalence of infections resistant to antibiotics.^30^ In our research, most isolates were susceptible to amikacin, kanamycin, streptomycin, gentamicin, fluoroquinolones, ceftiofur and colistin. Instead, greater resistant incidence rates were unveiled for amoxicillin, ampicillin, cefazolin, oxytetracycline, tetracycline, sulphadiazine, sulphamethoxydiazine and trimethoprim, which may be derived from the various, long-term and widespread use of these antimicrobials in the studied dairy farms. About 65.7% of *E. coli* isolates from CBM were resistant to more than 10 antimicrobial drugs. It is consistent with the findings that *E. coli* isolates from animals have been resistant to many antimicrobial agents used in clinics.^30^^,^^31^ Fluoroquinolones are potent antimicrobial agents for the treatment of various infections caused by *E. coli* in veterinary medicine. Unfortunately, fluoroquinolone-resistant *E. coli* in food-producing animals has been increasing in China because of the extensive usage of quinolones.^32^ We reported a similar occurrence (27.6%) of quinolone-resistant *E. coli* from BM cases, as compared with previous studies about dairy cattle suffering mastitis (22.9%) and endometritis (25.0%) in China.^1^ However, our result was much higher than data from developed countries, where the reported prevalence of quinolone-resistant *E. coli* in BM cases was 0% for both Canada and the United States.^33^^,^^34^ It may indicate that the widespread use of this class of antibiotics in dairy cows has contributed to a greater prevalence of quinolone resistance. Because the frequent occurrence of resistant isolates from food-producing animals would seriously threaten public health through the food chain, Chinese governmental agencies have taken measures to supervise antibiotic usage critically. Moreover, the use of quinolones in food-producing animals such as dairy cows had already been banned in China in 2015.^35^ Antimicrobial resistance in food-producing animals in China has been monitored since 2000. The resistance level of *E. coli* isolates from poultry to fluoroquinolones has increased by 50%.^36^ Previous studies on the resistance of *E. coli* to fluoroquinolones have been mainly based on data from swine and poultry.^37^ In 2008, Wang et al.^38^ reported that most *E .coli* isolates from BM were susceptible to fluroquinolones (70.69–77.59%). The resistance rates of these isolates for enrofloxacin, norfloxacin, lomefloxacin and ciprofloxacin were about 30%. In this study, the resistance rates of *E. coli* isolates from CBM to ciprofloxacin, lemofloxicin, norfloxacin and norfloxacin were 28.6%, 27.6%, 27.6% and 27.6%, respectively. The above results showed that the resistance of *E. coli* to fluoroquinolones did not increase in cows with mastitis. Although fluoroquinolones have been used for the treatment of different diseases in bovine and previously its prevalence was recorded between 20% and 30% in the past decade. The resistance of *E. coli* isolated from BM to fluoroquinolones drugs has always kept at a low level, which is closely related to rigorous measures taken by Chinese governmental agencies that all antimicrobials had been banned for animal growth promotion.

Generally, there are three major sources of MDR in BM such as; virulence factors, biofilm and some efflux pump activities.^39^ Some virulence factors such as haemolysins are responsible for the lysis of some immune cells of the host to invade and colonize into the host udder.^40^ In addition to this, the biofilm formation in the pathogenic organism acts as a protective layer against host immune response and antibiotic therapy.^41^ Finally, some efflux pump act as antibiotic resistance such as ESBLs, plasmid-mediated AmpC β-lactamases, carbapenemases and generalized efflux pump activity, but the RND-based tripartite efflux pump AcrAB-TolC is considered as most important factor for the antibacterial drug resistance.^42^

Fluoroquinolone resistance in *E. coli* is mostly linked to specific mutations in the QRDRs of the *gyrA* and *parC* genes of DNA topoisomerase II (DNA gyrase) and topoisomerase IV.^43^ These alterations may change how hydrogen bonds are formed, and it appears that interactions between quinolones and DNA are influenced by the amino acids’ negative charges complicate DNA-gyrase.^44^ The results of the study showed that these amino-acid substitutions Ser83Leu and Asp87Asn in the QRDR of *gyrA* were detected in 60.0% of *E. coli* isolates from CBM in China, which were in line with previous studies that reported point mutations in quinolone-resistant *E. coli* occurred more frequently in S83L/D87N in *gyrA.*^45^^,^^46^ The results of the study conducted by BANSAL also demonstrated utmost substitutions related to quinolone resistance ensue in the QRDR at the serine (Ser83) and aspartate (Asp87) *gyrA* protein’s residues in *E. coli* isolates.^47^ In our earlier research findings, we primarily noticed the substitutions in terms of amino acid positions Ser83Leu, Asp87Asn, Ser83Leu and Asp87Asn substitutions in *gyrA*, which are positioned within the QRDR.

In our research trial, in the QRDR of the *parC* protein different five amino-acid substitutions were found in 15 fluoroquinolone-resistant *E. coli* isolates from CBM, such as Arg138Asp, Ser58Ile, Pro140Thr, Arg138Val and Asn141Lys. The results indicated fluoroquinolone-resistance arises initially through mutations of g*yrA*, and additional mutation of *parC* leads to highly resistant *E. coli* isolates from cattle mastitis cases in China.

This was in line with past research findings that mutations targeting the DNA gyrase (topoisomerase II) were the main cause of fluoroquinolone resistance, with mutations in the topoisomerase IV causing higher levels of resistance.^48^ As reported by Sandhya Bansal,^48^ DNA gyrase (GyrA and GyrB) and topoisomerase IV (ParC and ParE) are the two essential type II topoisomerases. These enzymes act *via* inhibition of DNA replication. Alteration of a single amino acid at Ser-83 in GyrA is sufficient to generate decreased susceptibility to ciprofloxacin. Moreover, the accumulation of amino acid changes in GyrA and the simultaneous presence of ParC alterations contribute to high-level resistance to ciprofloxacin.

So, further, *parC* mutations are supposed to play a crucial role in highly resistant strain formation.^43^ Sáenz et al.^49^ previously stated that in GyrA a single amino acid exchange was deficient to confer a greater fluoroquinolone resistance phenotype in *E. coli* isolates. It was the same as our study’s conclusion that *gyrA* and *parC* mutations may contribute to the greater level of quinolone resistance in *E. coli* isolates from CBM in China. Moreover, in our study, all fluoroquinolone-resistant isolates with greater levels of ciprofloxacin resistance (≥32 µg/mL) carried double mutations in *gyrA* combined with one to three mutations in *parC*. These findings were in line with earlier findings that in the QRDR total point of mutations have been related to upsurge levels of fluoroquinolone-resistance.^22^^,^^47^

In this research trial, no PMQR determinants were noticed in all fluoroquinolone-resistant isolates. A similar report regarding PMQR genes had been published in a previous study.^1^ However, the results of the study done by Yang et al. displayed that the dominant PMQR genes among fluoroquinolone-resistant strains were *aac(6′)-Ib-cr* (44.0%) and *oqxA/B* (24.0%). In contrast, *qepA*, *qnrB* and *qnrS* were detected with lower frequencies.^9^ The results of this study revealed that, despite the growing interest in and reporting of PMQR mechanisms, the reduced susceptibility to quinolones in all *E. coli* isolates from CBM in China was caused by chromosomal mutations in the QRDR region rather than by plasmid-mediated quinolone resistance genes.

In this study, the prevalence of ESBLs in quinolone-resistant *E. coli* isolates was 100%. Our results showed that all quinolone-resistant *E. coli* isolates were recognized as β-lactamase producers. The predominant β-lactamase gene in the fluoroquinolone-resistant isolates was *bla*_TEM_, followed by *bla*_CTX_*_,_* and the *bla*_SHV_ was not detected. Our results did not align with previous studies that confirmed *bla_CTX-M_* was the utmost common β-lactams in *E. coli* from dairy cattle mastitis.^50^^,^^51^ In this study, the positive-integron incidence rate in *E. coli* isolates from CBM is 30.50%, which is lower than the data for the Beijing area of China, which was reported as 56.9%.^38^ Different studies reported that the ESBLs and integron prevalence are linked to various antibiotic pressures imposed by the intensive use of antibiotics in different environments. The results of this research showed that β-Lactamase genes *bla*_TEM_ and *bla*_CTX_ are frequently found together with integrons in fluoroquinolone-resistant isolates.^1^^,^^29^ The fact that ESBL genes could be acquired by strains harbouring particular integrons may increase the possibilities of selection of these isolates by a variety of different antimicrobials. Moreover, ESBL genes can be located on integrons, which may facilitate the spread of such genetic elements.^52^

## Conclusion

The results of the study showed a total of 220 cows with clinical mastitis were selected from 5000 cows. The incidence rate of CBM was 4.4% in current research. The isolates of *E. coli* from CBM were resistant to sulphonamides, tetracyclines, trimethoprim and penicillin, but most of them were susceptible to colistin, amikacin, ceftiofur and fluoroquinolones. Mutations in chromosomal genes encoding gyrase and topoisomerase IV are the main mechanisms responsible for high-level fluoroquinolone resistance and plasmid-mediated quinolone resistance (PMQR) have not been identified. Lastly, the findings of this study identified the prevalence of class I integrons and β-Lactamase genes in *E. coli* isolates from dairy cattle with CBM in different farms of the around Hohhot Inner Mongolia China. The isolates were positive for the class I integrase gene along with seven gene cassettes that were accountable for resistance to trimethoprim, aminoglycosides and chloramphenicol, respectively. The isolates that carried the *bla*_TEM_ and *bla*_CTX_ genes were resistant to penicillin, aminoglycosides, sulphonamides, trimethoprim, tetracyclines, chloramphenicol and fluoroquinolones. Since MDR and potential pathogenicity of *E. coli* is a serious problem among dairy cows in China, we predict that our findings will beneficial for relevant discussions to guide the urgent development of surveillance approaches to help control this phenomenon.

## Ethics approval and consent to participate

The methods and all the protocols used in this research were permitted by the related rules and regulations of the scientific research academic ethics committee, Inner Mongolia Agricultural University ([2020]086) that approved the study.

## Consent for publication

Not applicable

## Data Availability

Upon reasonable request, the corresponding author will provide all documents, data, and protocols that support the study’s findings. The links and accession numbers of the target genes are as follows: *gyrA* (https://www.ncbi.nlm.nih.gov/nuccore/X06373.1/;X06373), *parC* (https://www.ncbi.nlm.nih.gov/nuccore/M58408.1/;M58408), *GyrB* (https://www.ncbi.nlm.nih.gov/nuccore/AE000447.1/; AE000447), *ParE* (https://www.ncbi.nlm.nih.gov/nuccore/M58409.1/; M58409).
